# *CYP1A1* methylation mediates the effect of smoking and occupational polycyclic aromatic hydrocarbons co-exposure on oxidative DNA damage among Chinese coke-oven workers

**DOI:** 10.1186/s12940-019-0508-0

**Published:** 2019-07-29

**Authors:** Yanli Liu, Xuejing Li, Bin Zhang, Ye Fu, Aimin Yang, Hongjie Zhang, Huitao Zhang, Yingying Niu, Jisheng Nie, Jin Yang

**Affiliations:** 1 Department of Occupational Health, School of Public Health, Shanxi Medical University, Taiyuan, Xinjiannan Road 56, Taiyuan, 030001 Shanxi China; 2 Department of Preventive Medicine, School of Public Health and Management, Hubei University of Medicine, Shiyan, Hubei China; 3 Hong Kong Institute of Diabetes and Obesity, the Chinese University of Hong Kong, Hong Kong SAR, People’s Republic of China

**Keywords:** Oxidative DNA damage, Polycyclic aromatic hydrocarbons, Smoking, Cytochrome P4501A1, DNA methylation

## Abstract

**Background:**

Multiple factors, including co-exposure between lifestyle and environmental risks, are important in susceptibility to oxidative DNA damage. However, the underlying mechanism is not fully understood. This study was undertaken to evaluate whether Cytochrome P4501A1 (*CYP1A1*) methylation can mediate the co-exposure effect between smoking and occupational polycyclic aromatic hydrocarbons (PAH) in development of oxidative DNA damage.

**Methods:**

We explored the associations between smoking and occupational PAH co-exposure effect, *CYP1A1* methylation and oxidative DNA damage among 500 workers from a coke-oven plant in China. Urine biomarkers of PAH exposure (1-hydroxypyrene, 1-OHP; 2-hydroxynaphthalene, 2-NAP; 2-hydroxyfluorene, 2-FLU; and 9-hydroxyphenanthren, 9-PHE) and a marker of oxidative DNA damage (8-hydroxy- 2′- deoxyguanosine, 8-OHdG) were measured by high performance liquid chromatography. *CYP1A1* methylation was measured by pyrosequencing. Finally, mediation analysis was performed to investigate whether *CYP1A1* methylation mediated smoking and occupational PAH co-exposure effect on oxidative DNA damage.

**Results:**

We observed significant associations of smoking and 1-OHP co-exposure with *CYP1A1* hypomethylation (OR: 1.87, 95% CI: 1.01–3.47) and high 8-OHdG (OR: 2.13, 95% CI: 1.14–3.97). There was a significant relationship between *CYP1A1* hypomethylation and high 8-OHdG (1st vs. 3rd tertile = 1.58, 95% CI: 1.01–2.47, *P* for trend = 0.046). In addition, mediation analysis suggested *CYP1A1* hypomethylation could explain 13.6% of effect of high 8-OHdG related to smoking and 1-OHP co-exposure.

**Conclusions:**

Our findings suggested that the co-exposure effect of smoking and occupational PAH could increase the risk of oxidative DNA damage by a mechanism partly involving *CYP1A1* hypomethylation.

**Electronic supplementary material:**

The online version of this article (10.1186/s12940-019-0508-0) contains supplementary material, which is available to authorized users.

## Introduction

Oxidative DNA damage induced by reactive oxygen species (ROS) plays a pivotal role in the nosogenesis of respiratory disease such as lung cancer and asthma [1, 2]. ROS, resulting from chemical compounds or the action of exogenous physical factors such as ultraviolet A, or the metabolism of cells, can induce a great many DNA damage, including base modification and DNA strand breaks [3], were generally believed to be involved in the carcinogenic mechanism [4, 5]. Previous studies have shown that 8-hydroxy- 2′- deoxyguanosine (8-OHdG) is a widely accepted biomarker for assessing the extent of oxidative damage to DNA [6]. Lifestyle - environmental factors, such as smoking [7–9] and polycyclic aromatic hydrocarbons (PAH) exposure [10, 11], have been proved to relate with urine 8-OHdG levels.

Smoking has a strong effect on oxidative DNA damage, and PAH exposure is also related to oxidative DNA damage among occupational workers and general population [11–14]. There is a dose-dependent relationship of smoking and PAH metabolites in the risk of oxidative damage to DNA [15]. However, a challenge remains to fully understand the molecular mechanism of lifestyle - environmental factors between smoking and occupational PAH co-exposure effect on oxidative DNA damage.

Epigenetic modifications, for example, DNA methylation,which can be influenced by lifestyle – environmental factors, may provide a possible biological link between risk factors and the disease. Cytochrome P4501A1 (CYP1A1) is answerable for PAH metabolism, which participated in the metabolic process of exogenous compounds via the excessive formation of ROS [16, 17], eventually lead to the oxidative DNA damage. It has also been demonstrated that CYP1A1 can be induced by PAH and cigarette consumption can influence the *CYP1A1* methylation levels [18]. Increased lung cancer risk has been associated with high *CYP1A1* expression and hypermethylation [19]. Therefore, itʼs imperative to research whether *CYP1A1* methylation could mediate effect of smoking and PAH co-exposure on the development of oxidative damage to DNA.

We hypothesized that the co-exposure effect of smoking and PAH was involved in the development of oxidative DNA damage via *CYP1A1* methylation. To prove our hypothesis, we carried out the research to evaluate the co-exposure effect between smoking and PAH exposure on *CYP1A1* methylation and oxidative damage to DNA among coke-oven workers in China, so as to estimate whether *CYP1A1* methylation is responsible for increased risk of oxidative DNA damage related to the co-exposure effect of smoking and PAH.

## Material and methods

### Study subjects

The basic demographic data was collected from a coke-oven plant in China by using a cross-sectional survey in 2014. 950 workers participated in the study. We restricted our analyses to who had worked for more than 1 year, and who were non-exposed to known mutagens, for example, chemotherapy and radiotherapy in the last three months. We excluded individuals who were missing with sufficient blood samples (*n* = 360), sufficient urine samples (*n* = 228), or demographic characteristics (*n* = 278). Thus, the final analytic sample was 500 participants, of whom 389 coke-oven workers prolonged exposed to PAH and other 111 water treatment workers in the same plant without exposure to PAH in the workshop.

Trained interviewers collected the information regarding sex, age, years of working, education, smoking and drinking status, central heating and occupational exposure history by a pre-tested questionnaire. Smokers were defined as those who smoked at least 1 cigarette every day and continuously more than six months, and drinkers were drank at least once a week on average and continuously more than six months. After signing the informed consent, every participant provided with venous blood (5 mL) and morning urine (20 mL). And the study was approved by the Medical Ethics Committee of the Shanxi Medical University.

### Determination of urine PAH metabolites

The morning spot urine samples were collected and freezed at − 80 °C until further processing. The concentrations of PAH metabolites, including 1-hydroxypyrene (1-OHP), 2-hydroxynaphthalene (2-NAP), 2-hydroxyfluorene (2-FLU), and 9-hydroxyphenanthrene (9-PHE) were measured using high performance liquid chromatography (HPLC, Shimadzu Corp, JPN) according to the previously described protocol [20–22]. Urine creatinine (Cr) was measured by spectrophotometry (SpectraMAx M2, Molecular Devices, USA). Valid urine concentrations of PAH metabolites were adjusted using urine Cr concentrations and are expressed as μg/mmol Cr. The mean recovery rate, coefficient of variation (CV), *R*-square and limit of detection (LOD) were 82.97–107.85%, 2.04–4.27, 0.9998–1, and 0.04–0.12 μg/L, respectively. The concentrations less than LOD were expressed with half a LOD value.

### Urine 8-OHdG measurement

Urine 8-OHdG was measured by HPLC - electrochemical detector according to Yuan et al. [23] manuscript. In brief, about 2 mL supernatant of urine was prepared to elute twice with 0.1 mol/L KH_2_PO_4_ (PH 6.0), evaporate and dissolve with 1 mL KH_2_PO_4_. Standard curves of urine 8-OHdG were run daily to identify and quantify the concentration of urine samples. Valid urine concentrations of 8-OHdG were adjusted using urine Cr concentrations and are expressed as mmol/mol Cr. The mean recovery rate, CV, *R*-square and LOD, were 81–105%, 3.1, 0.9998, and 7.0 nmol/L, respectively.

### *CYP1A1* methylation measurement

DNA was extracted from whole blood according to MagBead blood DNA kit (ComWin Biotech, Beijing, China). The purity and concentration of extracted DNA was detected using ultraviolet and visible spectrophotometer (Eppendorf, Hamburg, Germany). Then bisulfite conversion using EZ DNA methylation Kit (ZYMO Research, California, USA). We choose a 153 bp fragment from − 944 to − 792 in promoter region of *CYP1A1* and 5 CpGs sites as the regions and sites of interest of the converted DNA based on previously published literature on *CYP1A1* methylation (Tekpli, 2012). The converted DNA was amplified using TaKaRa EpiTaq HS reagent (Takara, Dalian, China) in 50 mL, which contained 0.25 mL TaKaRa EpiTaq HS, 5 mL 25 mM MgCl_2_, 5 mL 10× EpiTaq Polymerase Chain Reaction (PCR) Buffer, 6 mL dNTP Mixture, 2 mL 10 mM forward primer, 2 mL 10 mM reverse primer, 100 ng bisulfite DNA and distilled water. Primer sequences for more detailed information could be seen in Additional file 1: Table S1. The PCR program was 95 °C 30 s; 95 °C 5 s, 60 °C 30 s, 72 °C 30 s, 40 cycles. PCR product was integrated in Streptavidin Sepharose High Performance (GE Healthcare, Sweden) to be purified, washed, denatured and washed again. The washed PCR product was annealed to 0.4 mM of sequencing primer and pyrosequencing was performed using PyroMark Q96 ID System (Qiagen, Hilden, Germany). The methylation degree was expressed as proportion of cytosines that were 5-methylated.

During the experiment, the sample of coke oven workers and water treatment workers were arranged alternately. In addition, we set a bisulfite trearment control before the variable positions in dispensation order to judge whether bisulfite treatment completely, and internal controls using PyroMark Control Oligo (Qiagen, Hilden, Germany) to determine if an unexpected result is related to the reagents, to the PyroMark Vacuum Workstations, or to the assay. The peak height of bisulfite control is not more than 7% of the average single peak height. The lowest single peak height was greater than 25 Relative Luminous Unit. The reduction in peak height between samples prepared using the PyroMark Q96 Vacuum Workstation compared with PyroMark Control Oligo added directly to the PyroMark Q96 Plate Low should not be more than 20%. Each CpG site had a quality assessments bar. Only quality assessments of all CpG sites in a well gave “Passed” quality, the methylation results could be used. The methylation levels were obtained from triplicate experiments, and the standard deviation was not exceed 2% units.

### Statistical analysis

We used the median and interquartiles range to describe the basic characteristics and laboratory parameters for continuous variables, which were skewed normal distribution, and tested using Mann-Whitney U test. The data of categorical variables were presented as frequency and proportion and tested using *Chi-square* test. Correlations of each PAH metabolites and each *CYP1A1* methylation sites were explored by Spearman’s correlation. We evaluated the effect of smoking on urine PAH metabolites by calculating smoking contribution to PAH metabolite [defined as the *R*^*2*^ difference between models with and without smoking]; other covariates were sex, age, years of working, drinking status, education and central heating. Logistic regression was conducted to evaluate associations of smoking and PAH metabolites co-exposure with *CYP1A1* hypomethylation and high 8-OHdG. The cutoff points for *CYP1A1* hypomethylation and high 8-OHdG defined as the 50th percentile were equal to 3.03 and 204.05 mmol/mol Cr, respectively. Covariates were adjusted including sex, age, years of working, smoking and drinking status (yes or no), education (< 9, 9–12, > 12), central heating (yes or no). A logistic regression model and a test of linear trend was used to estimate associations between *CYP1A1* hypomethylation and high 8-OHdG. The test for trend across decreasing tertile of *CYP1A1* methylation were conducted by assigning the medians of average *CYP1A1* methylation in tertiles treated as a continuous variable. Finally, we run a mediation analysis to investigate whether *CYP1A1* hypomethylation mediated smoking and PAH metabolites co-exposure effect on high 8-OHdG levels. The detailed instruction of mediation analysis could be seen in Lin et al. [24]. The mediation macro in SAS 9.4 (SAS Institute Inc., Cary, NC): %mediate (data=, id=, outcome=, exposure=, intermed=, modprint = T, intmiss = F, notes = nonotes, covars=, modopt=, procopt=, extrav=, where=, *RR*^2^ = 1, debugdv = 1, surv = 0, type = 1). The mediation effect was evaluated using the mediation percentage. There were statistically significance for *P* value < 0.05.

## Results

### Essential information

Table 1 showed the essential information of occupational workers with low (*n* = 250) and high (n = 250) 8-OHdG levels. Among 500 individuals, there were no statistically differences in sex, education, occupation, and central heating. Workers with high 8-OHdG were more likely to be older, longer years of working, smokers and drinkers. There were increasing trends in urine PAH metabolites with the increasing of urine 8-OHdG levels. And the urine 2-NAP and Σ PAH levels were significantly differences between low and high 8-OHdG levels (*P* < 0.05). The detailed distributions of urine PAH metabolites could be found in Additional file 1 :Table S2. Urine PAH metabolites were related to each other (Additional file 1 :Table S3); *CYP1A1* methylation at each sites were correlated with each other (Additional file 1 :Table S4), and we used average *CYP1A1* methylation to represent the *CYP1A1* methylation at each site. Average *CYP1A1* methylation levels in workers with high 8-OHdG levels were significantly lower than in workers with low 8-OHdG levels (2.42 vs. 3.10).

**Table 1:** Essential information and laboratory parameters among 500 occupational workers

Variables ^a^	low 8-OHdG (≤ 204.05 mmol/mol Cr)	high 8-OHdG (> 204.05 mmol/mol Cr)	*P* ^b^
Sex			
Male	217 (86.8)	228 (91.2)	0.116
Female	33 (13.2)	22 (8.8)	
Age (years)	39 (32–45)	41 (37–46)	0.009
Years of working	20 (14–27)	22 (17–28)	0.007
Education (years)			
< 9	53 (21.1)	66 (26.4)	0.273
9–12	92 (36.8)	94 (37.6)	
> 12	105 (42.0)	90 (36.0)	
Occupation			
Coke oven worker	191 (76.4)	198 (79.2)	0.451
Water treatment worker	59 (23.6)	52 (20..8)	
Central heating			
yes	237 (94.8)	233 (93.2)	0.451
no	13 (5.2)	17 (6.8)	
Smoking status			
yes	94 (37.9)	132 (53.2)	0.001
no	154 (62.1)	116 (46.8)	
Drinking status			
yes	142 (56.8)	178 (71.2)	0.001
no	108 (43.2)	72 (28.8)	
1-OHP (μg/mmol Cr)	0.05 (0.03–0.09)	0.06 (0.04–0.10)	0.144
2-NAP (μg/mmol Cr)	0.65 (0.36–1.07)	0.79 (0.44–1.25)	0.007
2-FLU (μg/mmol Cr)	0.26 (0.17–0.46)	0.28 (0.19–0.43)	0.395
9-PHE (μg/mmol Cr)	0.08 (0.06–0.14)	0.09 (0.06–0.17)	0.856
ΣPAH (μg/mmol Cr)	0.27 (0.18–0.46)	0.34 (0.23–0.48)	0.014
*CYP1A1* methylation			
pos.1	2.95 (0.00–4.36)	2.43 (0.00–4.07)	0.129
pos.2	2.19 (0.00–3.47)	1.83 (0.00–3.49)	0.913
pos.3	4.83 (2.24–6.17)	3.92 (0.00–6.00)	0.055
pos.4	2.40 (0.00–3.94)	2.09 (0.00–3.51)	0.477
pos.5	2.52 (0.00–4.53)	1.43 (0.00–3.99)	0.060
average	3.10 (1.63–4.20)	2.42 (1.29–3.85)	0.031

Drinking status missing 4 values

^a^ Data were presented as n (%) or Median (25th - 75th)

^b^ *P*-values were calculated from Chi-square test for categorical variables and Mann-Whitney U test for numerical variables

### The co-exposure effect of smoking and urine PAH metabolites on *CYP1A1* hypomethylation

First, we tested the contribution rates of smoking on urine PAH metabolites, and found smoking accounted for 0.29% of the 1-OHP variance, 8.99% of the 2-NAP variance, 0.11% of the 2-FLU variance, 6.37% of the 9-PHE variance and 1.99% of the Σ PAH variance (Additional file 1 :Table S5). Because of the higher contribution rates of smoking on 2-NAP and 9-PHE, we chose 1-OHP and 2-FLU as biomarkers of urine PAH metabolites to explore the co-exposure effect of smoking and occupational PAH on *CYP1A1* hypomethylation and high 8-OHdG.

**Fig. 1:**
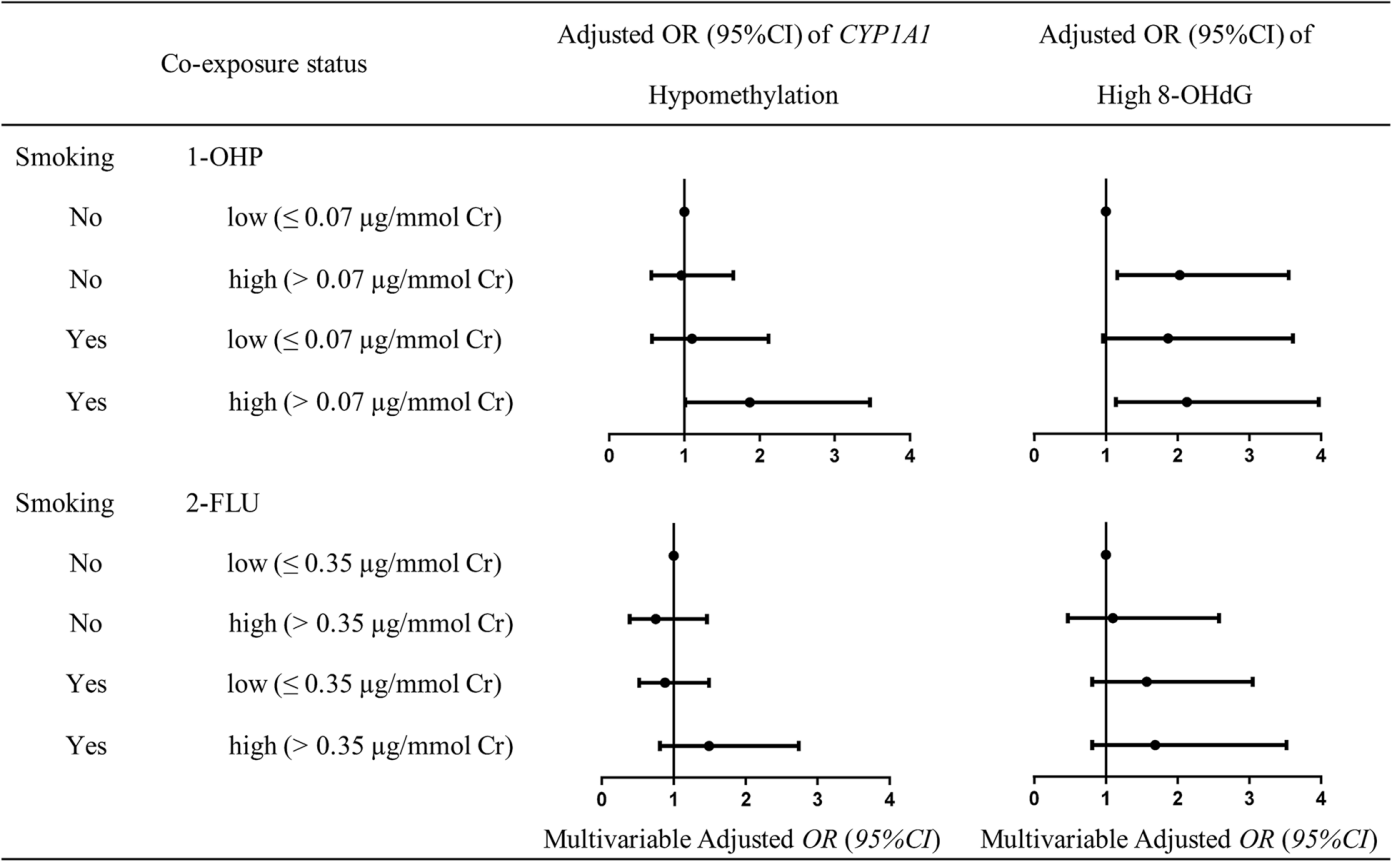
Co-exposure effects of smoking and urine PAH metabolites on risk of *CYP1A1* hypomethylation and high 8-OHdG. The statuses of smoking were stratified by non-smokers and smokers. The levels of urine PAH metabolites were stratified by the highest tertile into low exposure (< 67th percentile) and high exposure (≥ 67th percentile). The levels of *CYP1A1* methylation were stratified by the median (3.03) into *CYP1A1* hypomethylation (< 3.03) and *CYP1A1* hypermethylation (≥ 3.03). The levels of urine 8-OHdG were stratified by the median (204.05 mmol/mol Cr) into low 8-OHdG (< 204.05 mmol/mol Cr) and high 8-OHdG (≥ 204.05 mmol/mol Cr). Adjusted for sex, age, years of working, drinking status, education and central heating

The odds radios (ORs) and 95% confidence intervals (CIs) for the associations of smoking and 1-OHP co-exposure with *CYP1A1* hypomethylation were presented in Fig. 1. After adjusting covariates (i.e. sex, age, years of working, drinking status, education and central heating), we found that smoking and 1-OHP co-exposure was associated with *CYP1A1* hypomethylation (*P* < 0.05). That is, smokers who had high 1-OHP levels had about 1.87 (1.01–3.47) times risk of *CYP1A1* hypomethylation, compared to non-smokers who had low 1-OHP levels. The same increasing trends could be observed in smoking and 2-FLU co-exposure effects on *CYP1A1* hypomethylation levels, but were not significantly difference (*P* > 0.05).

### The co-exposure effect of smoking and urine PAH metabolites on high 8-OHdG

The ORs for association of smoking and urine PAH metabolites co-exposure with high 8-OHdG were presented in Fig. 1. After adjusting covariates, we observed smokers who with high 1-OHP levels had significantly higher 8-OHdG levels compared with non-smokers who with low 1-OHP levels [*OR* (95% *CI*): 2.13 (1.14–3.97)]. Smokers, no matter exposed to low or high levels of urine 2-FLU, had an increasing risk of high 8-OHdG levels compared with non-smokers.

### The association between *CYP1A1* hypomethylation and high 8-OHdG

The association between *CYP1A1* hypomethylation and high 8-OHdG could be found in Table 2. The risk of high 8-OHdG levels was on the rise trend when the *CYP1A1* methylation levels gradually decreased. The crude *OR*s (95%*CI*) of high 8-OHdG for decreasing tertile of *CYP1A1* methylation were 1.00 (reference), 1.24 (0.81–1.91), and 1.66 (1.08–2.57), respectively (*P*_trend_ = 0.021). In the lowest tertile of *CYP1A1* methylation, the *OR* of high 8-OHdG decreased to 1.58 (1.01–2.47) when adjusting for all covariates, with *P* for trend = 0.046.

**Table 2:** The association between *CYP1A1*hypomethylation and high 8-OHdG among 500 occupational workers

*CYP1A1* methylation	*n*	Crude OR (95%CI)	Adjusted ORa (95%CI)
Tertile1 (≤ 2.07)	166	1.66 (1.08–2.57)	1.58 (1.01–2.47)
Tertile2 (2.07–3.81)	168	1.24 (0.81–1.91)	1.24 (0.79–1.92)
Tertile3 (> 3.82)	166	1.00 (reference)	1.00 (reference)
*P* for trend		0.021	0.046

^a^Multiple logistic regression with adjusted for sex, age, years of working, drinking status, education and central heating

### *CYP1A1* hypomethylation mediated the co-exposure effect of smoking and urine PAH metabolites on high 8-OHdG

We performed mediation analysis of *CYP1A1* hypomethylation in the association between smoking and PAH metabolites co-exposure and oxidative DNA damage. We observed a significant mediation effect of *CYP1A1* hypomethylation in the association between smoking and 1-OHP co-exposure and high 8-OHdG in Table 3 (*P* = 0.047). The mediation analysis showed a mediation proportion of 13.6% (95% CI: 2.6–47.9%). These results suggested that *CYP1A1* hypomethylation may be a potential mediator of smoking and 1-OHP co-exposure effect on the risk of oxidative DNA damage. However, we didn’t find that the associations between smoking and 2-FLU co-exposure and high 8-OHdG were mediated by *CYP1A1* hypomethylation.

**Table 3:** *CYP1A1* hypomethylation mediated the effect of smoking and 1-OHP co-exposure on high 8-OHdG

Co-exposure	Total effect	Direct effect	Proportion mediated by *CYP1A1* hypomethylation	*P* value
Smoking and1-OHP	1.03 (1.00–1.06)	1.02 (1.00–1.05)	13.6% (2.6–47.9%)	0.047

Covariates in the SAS macro include sex, age,years of working, drinking status, education and central heating

^a^Total effects of smoking and 1-OHP co-exposure on high 8-OHdGwere estimated without adjusting for *CYP1A1*hypomethylation

^b^Direct effects of smoking and 1-OHP co-exposure on high 8-OHdG were estimated with adjusting for *CYP1A1*hypomethylation

## Discussion

In this study, we observed the co-exposure effect of smoking and 1-OHP were positively associated with *CYP1A1* hypomethylation and high 8-OHdG (a biomarker of oxidative damage to DNA) after adjusting for covariates. We also investigated a positive relationship between *CYP1A1* hypomethylation and high 8-OHdG in an upwardly trending, dose-responsive manner. Moreover, *CYP1A1* hypomethylation may serve as a potential mediator of smoking and occupational PAH co-exposure effect on risk of oxidative DNA damage.

In the current study, we also detected the concentration of environmental PAH, since the air PAH levels in the plant could represent the exposure status of occupational workers. The results showed the sum PAH of the workplace was markedly lower than Kuang et al. [11]: 0.38 mg/m^3^ vs. 1.13 mg/m^3^ in the non coke-oven place, 1.45 mg/m^3^vs. 11.08 mg/m^3^at the side of the coke-oven. The big difference of environmental PAH exposure may account for internal exposure different. PAH internal exposure, which showed various pathways of exposure, could be more accurate to reflect the actual levels of PAH exposure. Urinary 1-OHP was a widely used short-time biomarker of PAH exposure, and had a linear relationship with PAH concentration in the workplace [25]. But it alone cannot reflect the overall internal PAH metabolites, and urine hydroxylated nathalene [26], hydroxylated fluorene and hydroxylated phenanthrenes were suggested to be good surrogate biomarkers of occupational PAH exposure. Urine PAH metabolites were regarded as biomarkers to evaluate external PAH exposure [27]. The concentration of urine PAH metabolites in our research were lower than Kuang et al. [11] and Talaska et al.ʼs [28] researches. Besides external PAH exposure, regional differences, air pollution, lifestyle behaviors and laboratory methods can also cause the difference in urine PAH metabolites.

Some studies indicated smoking can alter the DNA methylation status [29–32]. Tekpli [18] reported smoking was associated with *CYP1A1* methylation. Other studies suggested PAH exposure was related to DNA methylation [33–35]. In our study, we observed smoking and 1-OHP co-exposure was associated with *CYP1A1* hypomethylation, further proved that smokers exposed to PAH were more likely to lower *CYP1A1* methylation levels, which were consisted with other studies. However, the underlying mechanisms of methylation changes resulted from smoking or PAH exposure remain unknown. As far as *CYP1A1* is concerned, we can speculate aryl hydrocarbon receptor (AHR) binding to *CYP1A1* promoter region accelerated after smoking or PAH exposure, may cause the *CYP1A1* methyltransferase removed from the promoter and subsequently lead to a loss of methylation. Therefore, the *CYP1A1* methylation could be inducted by gene expression, but could promote the binding of AHR and strengthen transcriptional activity in return [36].

PAH can be metabolized by CYP1A1, and then generate ROS, which were known to cause oxidative DNA modification. As one of the predominant forms of oxidative lesions in DNA, 8-OHdG is a specific and quantitative biomarker of oxidative damage to DNA [37, 38]. Urine 8-OHdG is highly affected by many factors, such as sex, age, smoking, occupational exposure, and so on. Some studies had showed PAH exposure was positively related to urine 8-OHdG whether in the occupational worker or general population [14, 39, 40]. Asami et al. [9] showed a significant relationship between the Brinkman index and 8-OHdG levels. Our study revealed smoking and 1-OHP co-exposure was positively related to high 8-OHdG levels, indicating smokers, which are occupational exposed to PAH in the long term, may have more serious oxidative damage to DNA. These results were consistent with the previously reported findings [15].

Even though the smoking and 1-OHP co-exposure plays an important role in the high 8-OHdG levels, a challenge remains to provide a functional interpretation and investigate the further mechanism of smoking and PAH exposure on the development of oxidative damage to DNA. Some studies reported that gene methylation was associated with DNA damage [10, 35, 41]. We also showed a dose-responsive relationship between *CYP1A1* hypomethylation and high 8-OHdG levels. These findings add to the strength of relationship between DNA methylation and oxidative DNA damage. DNA methylation, which can reflect the co-exposure between environmental factors, could be a possible “missing link” and is an attractive mechanism to explain the formation of oxidative damage to DNA. The mediation analysis suggested that 13.6% effect of oxidative DNA damage related to smoking and 1-OHP co-exposure was mediated by *CYP1A1* hypomethylation, indicating that smoking and PAH co-exposure may influence oxidative damage to DNA through *CYP1A1* hypomethylation. In fact, it has been established that *CYP1A1* expression is important in the metabolic process of xenobiotic and *CYP1A1* mRNA levels correlate with DNA damage levels [42, 43]. Since the gene methylation plays a pivotal role in regulating expression, a compellent viewpoint is the epigenetic regulation of *CYP1A1* expression may be an important factor in xenobiotic-related to oxidative DNA damage. The altered DNA methylation in promoter region, which may influence assembly and gene expression of *CYP1A1*, could influence PAH metabolic process in vivo, eventually lead to the oxidative DNA damage.

Nevertheless, our study also has some limitations. For the nature of cross-sectional, our results cannot establish a causal association between PAH exposure, *CYP1A1* hypomethylation and oxidative DNA damage. Besides, urine cotinine levels as a specific biomarker of smoking, body mass index, physical activity, eating habits, etc., which can affect the urine 1-OHP and 8-OHdG concentration, and white blood cell subtype, which may have an impact on DNA methylation, weren’t duly taken into account. Finally, the smaller sample size and relatively less biomarkers of PAH exposure are the shortages for our study. Nevertheless, we still observed the co-exposure effect of smoking and 1-OHP on oxidative DNA damage was partly mediated by *CYP1A1* hypomethylation. We didnʼt find that *CYP1A1* hypomethylation mediated the co-exposure effect of smoking and 2-FLU on oxidative DNA damage. One potential explanation is that the most representative metabolites of PAH exposure in urine were 1-OHP for occupational workers in coke oven plants. Many studies suggested that 1-OHP was a suitable PAH internal exposure biomarker in coke-oven workers [28, 44], which was further confirmed by our results. In addition, there may be other pathways involved to further research.

## Conclusions

In this study, we quantitative assessed the association of smoking and occupational PAH co-exposure with both *CYP1A1* hypomethylation and oxidative damage to DNA in Chinese occupational workers. We observed *CYP1A1* hypomethylation may partly mediate the co-exposure effect of smoking and occupational PAH on oxidative DNA damage for the first time. From the point of public health, further prospective researches are necessary.

## Additional file

Additional file 1: **Table S1.** Primers used for pyrosequencing size of the PCR amplicons and position of the primers from the transcription start point. **Table S2.** Distributions of urine PAH metabolites among 500 occupational workers. **Table S3.** The correlation coefficients (r_s_) of urine PAH metabolites among 500 occupational workers. **Table S4.** The correlation coefficients (*r*_*s*_) of *CYP1A1* methylation among 500 occupational workers. **Table S5.** Contribution rates of smoking on urine PAH metabolites among 500 occupational workers. (DOC 67 kb)

## Data Availability

The data that support the findings of this study are available from the corresponding author upon reasonable request.

## Abbreviations

**1-OHP**: 1-hydroxypyrene
**2-FLU**: 2-hydroxyfluorene
**2-NAP**: 2-hydroxynaphthalene
**8-OHdG**: 8-hydroxy-2′-deoxyguanosine
**9-PHE**: 9-hydroxyphenanthrene
**AHR**: Aryl hydrocarbon receptor
**CI**: Confidence interval
**Cr**: Creatinine
**CV**: Coefficient of variation
**CYP1A1**: Cytochrome P4501A1
**LOD**: Limit of detection
**PAH**: Polycyclic aromatic hydrocarbons
**PCR**: Polymerase Chain Reaction
**ROS**: Reactive oxygen species
