# Supplementary material for: CYP1A1 methylation mediates the effect of smoking and occupational polycyclic aromatic hydrocarbons co-exposure on oxidative DNA damage among Chinese coke-oven workers
Source: Environ Health. 2019 Jul 29;18:69. doi: 10.1186/s12940-019-0508-0 (PMC6664755; doi:10.1186/s12940-019-0508-0)
Supplement: Supplementary file 1 — Table S1. Primers used for pyrosequencing size of the PCR amplicons and position of the primers from the transcription start point. Table S2. Distributions of urine PAH metabolites among 500 occupational workers. Table S3. The correlation coefficients (rs) of urine PAH metabolites among 500 occupational workers. Table S4. The correlation coefficients (rs) of CYP1A1 methylation among 500 occupational workers. Table S5. Contribution rates of smoking on urine PAH metabolites among 500 occupational workers. (DOC 67 kb) [file 12940_2019_508_MOESM1_ESM.doc]

**SUPPLEMENTAL INFORMATION**

***CYP1A1* methylation mediates smoking and occupational polycyclic aromatic hydrocarbons co-exposure effect on oxidative DNA damage among Chinese coke-oven workers**

**Yanli Liu, Xuejing Li, Bin Zhang, Ye Fu, AiminYang, Hongjie Zhang, Huitao Zhang, Yingying Niu, Jisheng Nie, Jin Yang**

| **Table S1. Primers used for pyrosequencing size of the PCR amplicons and position of the primers from the transcription start point.** | | | |
| --- | --- | --- | --- |
| Gene | Primers | Position | Size |
| *CYP1A1* | F: 5´ ATG GGT AGG TTG GGT GGT T 3´  R: 5´ (biotin)ACC CTA CCC CCC ACT CTA ACT TAC 3´  S: 5´ GTA GGT TGG GTG GTT G 3´ | -944 bp  -792 bp  -940 bp | 153 bp |

| **Table S2. Distributions of urine PAH metabolites among 500 occupational workers** | | | | | | |
| --- | --- | --- | --- | --- | --- | --- |
| Urine PAH metabolites (μg/mmol Cr) | Mean (95%CI) | Percentiles | | | | |
|  |  | 5th | 25th | 50th | 75th | 95th |
| 1-OHP | 0.10 (0.09 - 0.12) | 0.02 | 0.03 | 0.05 | 0.09 | 0.36 |
| 2-NAP | 0.87 (0.81 - 0.93) | 0.18 | 0.39 | 0.72 | 1.16 | 2.11 |
| 2-FLU | 0.40 (0.36 - 0.44) | 0.10 | 0.19 | 0.28 | 0.44 | 1.12 |
| 9-PHE | 0.14 (0.12 - 0.15) | 0.04 | 0.06 | 0.09 | 0.15 | 0.41 |
| ΣPAH | 0.38 (0.35 - 0.40) | 0.11 | 0.20 | 0.30 | 0.47 | 0.89 |

| **Table S3. The correlation coefficients (r_s_) of urine PAH metabolites among 500 occupational workers** | | | | | |
| --- | --- | --- | --- | --- | --- |
| *r_s_* | 1-OHP | 2-NAP | 2-FLU | 9-PHE | Σ PAH |
| 1-OHP | 1.00 | 0.30^*^ | 0.59^*^ | 0.61^*^ | 0.54^*^ |
| 2-NAP |  | 1.00 | 0.46^*^ | 0.09^#^ | 0.88^*^ |
| 2-FLU |  |  | 1.00 | 0.63^*^ | 0.77^*^ |
| 9-PHE |  |  |  | 1.00 | 0.43^*^ |
| ΣPAH |  |  |  |  | 1.00 |
| ** P* < 0.001, ^#^ *P* < 0.05 | | | | | |

| **Table S4. The correlation coefficients (*r_s_*) of *CYP1A1* methylation among 500 occupational workers** | | | | | | |
| --- | --- | --- | --- | --- | --- | --- |
| *r_s_* | pos.1 | pos.2 | pos.3 | pos.4 | pos.5 | average |
| pos.1 | 1.00 | 0.36^*^ | 0.40^*^ | 0.36^*^ | 0.23^*^ | 0.64^*^ |
| pos.2 |  | 1.00 | 0.43^*^ | 0.49^*^ | 0.41^*^ | 0.67^*^ |
| pos.3 |  |  | 1.00 | 0.50^*^ | 0.45^*^ | 0.81^*^ |
| pos.4 |  |  |  | 1.00 | 0.55^*^ | 0.74^*^ |
| pos.5 |  |  |  |  | 1.00 | 0.69^*^ |
| average |  |  |  |  |  | 1.00 |
| ** P* < 0.001 | | | | | | |

| **Table S5. Contribution rates of smoking on urine PAH metabolites among 500 occupational workers** | | | | | | | |
| --- | --- | --- | --- | --- | --- | --- | --- |
| urine PAH metabolites | Model 1^a^ | |  | Model 2^b^ | |  | Variance of urine PAH metabolites  explained by smoking ^c^ |
|  | *R^2^* | *p* |  | *R^2^* | *p* |  | Δ*R^2^* (*%*) |
| 1-OHP | 0.044 | 0.181 |  | 0.047 | 0.026 |  | 0.291 |
| 2-NAP | 0.147 | 0.546 |  | 0.237 | 0.571 |  | 8.990 |
| 2-FLU | 0.049 | 0.315 |  | 0.050 | 0.072 |  | 0.109 |
| 9-PHE | 0.037 | 0.631 |  | 0.100 | 0.603 |  | 6.374 |
| ΣPAH | 0.108 | 0.397 |  | 0.127 | 0.594 |  | 1.985 |
| ^a^ Model 1: Binary logistic regression models with urine PAH metabolite as dependent variables, sex, age, years of working, drinking, education, central heating included as independent variables.  ^b^ Model 2: additionally included smoking in Model 1.  ^c^ The variance of each urine PAH metabolite explained by smoking, calculated as the variance explained by Model 2 minus the variance explained by Model 1. | | | | | | | |
